# Examining the effect of explanation on satisfaction and trust in AI diagnostic systems

**DOI:** 10.1186/s12911-021-01542-6

**Published:** 2021-06-03

**Authors:** Lamia Alam, Shane Mueller

**Affiliations:** Michigan Technological University, Houghton, MI 49931 USA

## Abstract

**Background:**

Artificial Intelligence has the potential to revolutionize healthcare, and it is increasingly being deployed to support and assist medical diagnosis. One potential application of AI is as the first point of contact for patients, replacing initial diagnoses prior to sending a patient to a specialist, allowing health care professionals to focus on more challenging and critical aspects of treatment. But for AI systems to succeed in this role, it will not be enough for them to merely provide accurate diagnoses and predictions. In addition, it will need to provide explanations (both to physicians and patients) about why the diagnoses are made. Without this, accurate and correct diagnoses and treatments might otherwise be ignored or rejected.

**Method:**

It is important to evaluate the effectiveness of these explanations and understand the relative effectiveness of different kinds of explanations. In this paper, we examine this problem across two simulation experiments. For the first experiment, we tested a re-diagnosis scenario to understand the effect of local and global explanations. In a second simulation experiment, we implemented different forms of explanation in a similar diagnosis scenario.

**Results:**

Results show that explanation helps improve satisfaction measures during the critical re-diagnosis period but had little effect before re-diagnosis (when initial treatment was taking place) or after (when an alternate diagnosis resolved the case successfully). Furthermore, initial “global” explanations about the process had no impact on immediate satisfaction but improved later judgments of understanding about the AI. Results of the second experiment show that visual and example-based explanations integrated with rationales had a significantly better impact on patient satisfaction and trust than no explanations, or with text-based rationales alone. As in Experiment 1, these explanations had their effect primarily on immediate measures of satisfaction during the re-diagnosis crisis, with little advantage prior to re-diagnosis or once the diagnosis was successfully resolved.

**Conclusion:**

These two studies help us to draw several conclusions about how patient-facing explanatory diagnostic systems may succeed or fail. Based on these studies and the review of the literature, we will provide some design recommendations for the explanations offered for AI systems in the healthcare domain.

**Supplementary Information:**

The online version contains supplementary material available at 10.1186/s12911-021-01542-6.

## Introduction

### Background

AI systems are increasingly being fielded to support diagnoses and healthcare advice for patients [1]. Although these systems are still in their infancy, they have the potential to serve as a first point-of-contact for patients, and eventually may produce diagnoses and predictions about patient’s health, perform routine tasks, and provide non-emergency medical advice. This has the potential to provide innovative solutions for improved healthcare outcomes at a reduced cost. In fact, numerous systems are currently in development or being fielded that place an AI as the first point of contact for patients [2–13]. Almost all these systems (e.g., chatbots) are dialogue-based and provide initial diagnosis, medical advice, or consultation based on the information they gather from the users. Some of them may also provide recommendations if the patient needs to visit the doctor or submit report to doctors for further analysis. These systems have the potential to address the efficiency gap and thus reduce medical costs by automating tasks, triaging patients to the most appropriate services and allowing them to self-care, and thus have enthusiasm from medical providers. For example, the UK’s National Health Services (NHS) announced a partnership with Amazon to allow elderly people, blind people and other patients who cannot easily search for health advice on the internet to access the information through the AI-powered voice assistant Alexa [13]. That day is not far when AI might take a primary role in the initial consultation, routine check-ups, or triage advice. However, in order to replace or supplement human diagnosis from physicians and health care professionals, it may not be enough for the AI diagnosis system to just be accurate. An accurate diagnosis without justification or explanation might be ignored, even from a competent physician. This was perhaps first noted in the early days of medical diagnosis systems, Teach and Shortliffe [14] found that when considering AI diagnostic systems, the most important desire of both physicians and non-physicians was that it should be able to explain its diagnostic decisions. In contrast, avoiding incorrect diagnoses and erroneous treatments were rated among the least important properties. A more recent study surveying expert physicians on clinical decision support systems (CDSS) [15] showed that although accuracy is now a greater concern, understanding the underlying reasoning of the system (“CDSSs act like block boxes”) remains a top concern among diagnosticians. Other research shows that clinicians' trust and understanding of AI diagnostic systems are also improved by explainable systems [16, 17]. Recently, Holzinger et al. [18] argued that Explainable AI (XAI) may help to facilitate transparency and trust for the implementation of AI in the medical domain, so we expect that any successful patient-focused AI diagnoses system will also provide explanations and justifications of that diagnosis so that the patient can understand why a diagnosis is made or a treatment plan is recommended. It is even possible that an average diagnosis system with better explanation will lead to better healthcare outcomes than a perfect diagnosis system without explanation.

A variety of algorithms have been identified for providing explanations of AI diagnostic systems, both within and outside the field of healthcare. For example, early expert systems provided rule-based logical explanations that were tightly coupled to the knowledge the systems used to make diagnoses [19–23]. More recently, researchers have focused on visualizing elements of the classification algorithms being used to make a diagnosis (e.g., heat-map image analysis), and visualizing decision trees or complex additive models [24–28]. Other researchers have explored using case-based explanations, providing examples, and compelling support for the systems’ conclusions [29–35]. Consequently, there are several algorithmic approaches to both diagnosis and explanation of diagnoses that have been explored in medical AI. However, neither of these approaches has emphasized on patient-centered communication which helps to develop a shared understanding of patient problems [36]. Patient-centered communication considers a patient as an active partner in the healthcare environment and values the patient’s wants, needs, and preferences [37]. Explanations to the patients are a crucial part of the communication [38] and it helps to understand their illness and problems in a clearer way [39]. Physicians who exhibit these behaviors gain a higher level of trust among patients [40]. A recent study also showed that if patients feel that the AI systems are providing them personalized care, it helps reduce the resistance to AI-based diagnosis [41]. But it is not clear which current XAI methods are effective for patient-centered communication, whether a single method is sufficient, or whether the explanations need to be tailored to individual patients, situations, or different timepoints during diagnosis. Furthermore, the literature on XAI in healthcare focuses primarily on algorithms, rather than the impact the algorithms have on patients (such as their satisfaction, trust, understanding, or willingness to use the system in the future) for establishing their compatibility with patient-centered communication [42]. Understanding how trust in these systems is constructed would provide an insight into how these systems would be used, misused, or disused [43].

One approach we have pursued to study explainable AI (XAI) in healthcare is to understand the types of explanations real physicians offer when they interact with patients. For example, Alam [44] conducted an interview study with physicians to document how they explained diagnoses to the patients. The results suggest that physicians use a variety of explanation methods, which are dependent on context, including time (i.e., early or later in diagnosis) and the patient or patient’s advocate’s identity (including cultural, education, age, and other concerns). The explanations identified included the use of logical arguments, examples, test results, imagery, analogies, and emotional appeals. The results of this study also suggest that physicians tend to provide different types of explanations at different points of diagnosis. Although many of these explanations have been explored in the XAI literature [45], few systems have acknowledged the variety and contextual aspects of the different explanation types.

### Methods for providing explanations

In the present paper, we will report on two experiments we conducted that explore how different types of explanations may impact satisfaction and trust in a simulated AI diagnostic system. In these studies, we will examine several different types of explanations that have been proposed and explored in the XAI community. As such, our explanations are general and might apply to many kinds of AI and Machine Learning algorithms, from rule-based systems, Bayes networks, and decision trees to deep networks and complex ensemble approaches. One such type of explanation is whether the goal of the explanation is to inform about the diagnostic process, versus justify why a particular diagnosis was made. These explanation types are respectively referred to as “global” and “local” explanations [46–49].In general, Alam [44] found that physicians report using both methods; sometimes they explain how a particular disease or diagnostic process works; other times they justify why a particular diagnosis is given based on evidence (symptoms, test results, history, etc.).

Another important distinction is the means by which an explanation is provided. Alam [44] also found that physicians’ explanations mapped onto many of the explanation types studied in the XAI literature, including case-based information and examples [50, 51], analogies [52, 53], logical arguments [54, 55], visualizing imagery and highlighting important aspects [56, 57]. For imagery, AI healthcare systems may use graphs to show the relative probability of different outcomes or the relative importance of different symptoms for those outcomes, which is more akin to how the LIME algorithm [58] works for diagnostic features. Physicians may present visualization differently from how AI systems offer visual explanations, but even the use of x-rays and other test reports are generally accompanied by explanations highlighting the location of critical signs indicating a diagnosis—with a similar goal as gradient-based heatmaps [59–62] in XAI systems. Of course, the particular visualizations provided by algorithms such as LIME [63] may themselves be hard to understand. We are focused on the general question of whether representing feature and outcome importance is informative, and not the specific question of whether a particular algorithm that supports this is useful.

Next, we will report the results of two studies in which we tested a variety of explanation methods and approaches in a simulated diagnostic situation. Rather than testing a single explanation of an isolated case, we designed a garden path scenario in which symptoms initially pointed to one diagnosis, but later it became clear that another diagnosis was correct. This provided an emerging diagnosis, which we believe is particularly well-suited to understand how simulated patients both trust and understand an AI diagnostic system.

## Experiment 1

Hoffman et al. [64] argued that elements of satisfaction and trust follow from an improved understanding of an AI system that might be gathered from different kinds of explanations. Consequently, we hypothesize that explanations will induce greater satisfaction, trust, understanding, and perceptions of accuracy. However, no existing theories suggest whether these benefits should exist throughout a scenario, or only at certain time points, and so we will investigate whether these potential benefits change over time. We also hypothesize that both global and local explanations will be beneficial in comparison to control but may have differential effects when compared to one another. To investigate this, we tested participants interacting with a simulated AI system that initially gives the most likely but incorrect diagnosis, but later it changes the diagnosis to the correct disease once further testing is complete. This provides an important case for understanding explanation, because, at all times, the AI can be judged to be behaving optimally given its information—even when its diagnosis is incorrect.

### Method

#### Participants

Eighty undergraduate students at Michigan Technological University took part in the study in exchange for partial course credit. They were enrolled in the “Introductory to Psychology” course. Students in the class are typically first or second-year undergraduate students.

#### Procedure

We created a diagnosis scenario in which a simulated AI system gives a most likely but incorrect diagnosis but later changes the diagnosis to the correct disease. The scenario involved gastrointestinal disorders and symptoms, which are often difficult to diagnose in real-world situations. The participants played the role of patients in the scenario, instructed to say they were suffering from specific symptoms (abdominal pain, cramps, diarrhea, fatigue, and joint pain). A simulated AI system (called MediBot.ai) provided diagnostic information about the scenario, initially concluding that the patient was suffering from Irritable Bowel Syndrome (IBS), and advised patients to follow a specific diet chart and come back for follow up next week. After one week, the participants were told that they had begun to feel better, but the symptoms started getting worse after that. When the patient did not feel good even after three consecutive weeks, MediBot determined that the patient might not be suffering from the “most likely” condition IBS and changed its diagnosis, ordered additional diagnostic tests, and determined the patient was suffering from Celiac disease, which occurs due to gluten allergy (the ‘ground truth’ of the scenario). Participants had to communicate with MediBot through six simulated weeks, but the study took around 20 min to complete. All participants experienced the same basic scenario with identical symptoms and diagnoses. To maintain certain intervals between the simulated weeks, they were given brief crosswords to solve during the intervals. After they solved one crossword, they were asked to start following up with MediBot and play their role as patients again.

**Fig. 1:**
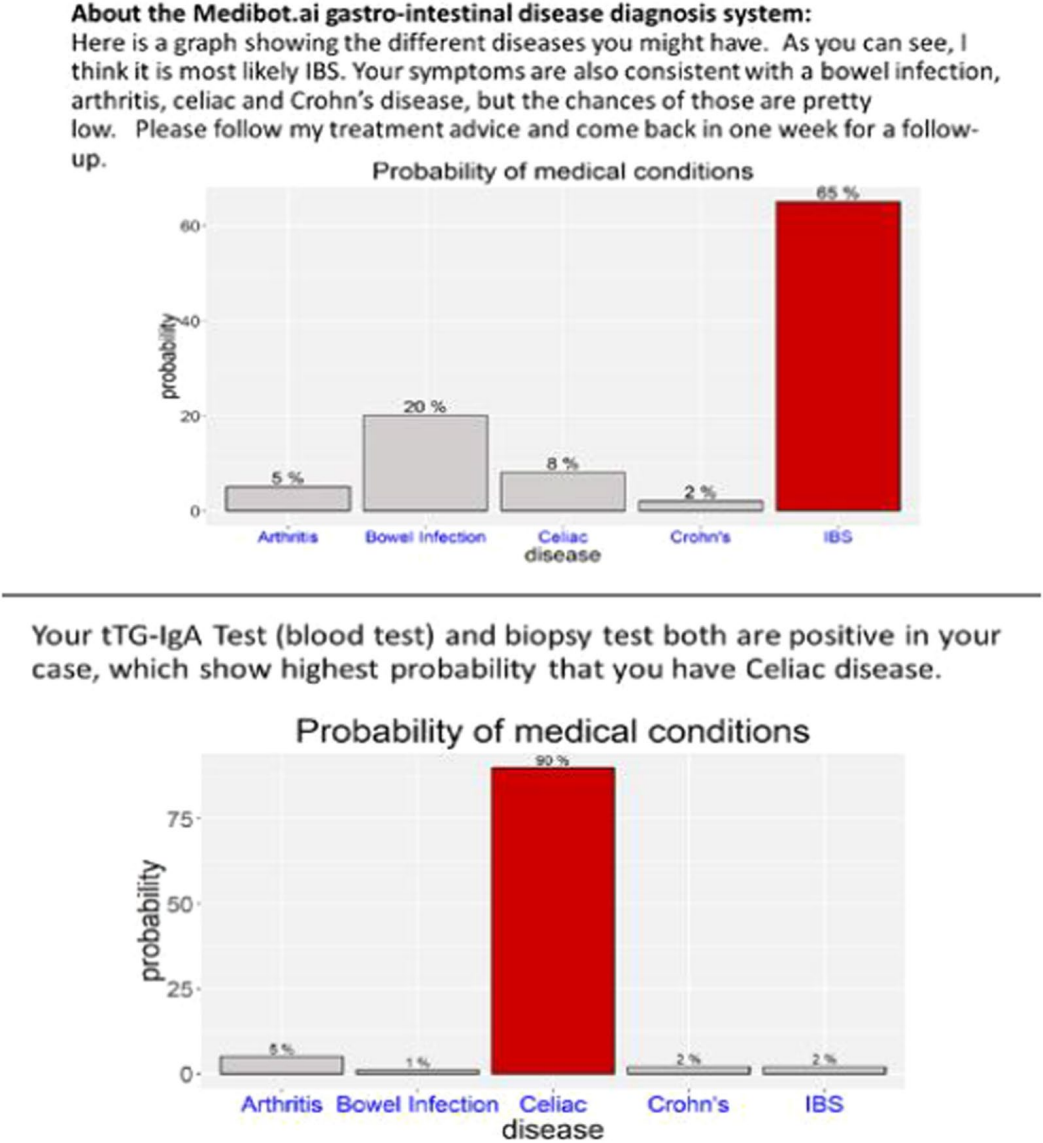
Week 4 (top panel) and 5 (bottom panel) Probability Chart and Explanation. Initial diagnosis of IBS changes as information emerges, and the explanations constitute the relative certainty of each disease in these bar charts and text description

Participants were divided into three groups: Control, global explanation, and local explanation. The control group received no explanation of why MediBot was making any decision in any week. The global explanation group received an initial tutorial describing how MediBot does diagnosis in general and focusing especially on how the AI follows a most-likely diagnostic approach, which means that it may make errors in particular cases. This included two examples: (1) A success case of the first diagnosis, and (2) a failure case for the first diagnosis, but eventually a successful second diagnosis. The Local explanation group received local justifications about each decision and prediction of MediBot throughout the scenario. Local justifications explained why the MediBot made a particular decision for a particular case. For this group, MediBot showed a probability chart of the disease likelihood of the patient in each week (see Fig. 1), representing the probability or likelihood of different outcomes visually, and including descriptive text explanation about why it was making a particular decision.

The Additional file 1: Appendix lists the entire scenario for a patient across six weeks of diagnosis. After each simulated week, participants were asked to rate their satisfaction, trust, perception of accuracy, sufficiency, usefulness, and completeness for the explanations received from MediBot (Rating scale range was 1–21), These are some of the key attributes of explanations identified in the literature and are referred to as “Explanation Satisfaction Scale” attributes [64]. At the end of the study, participants also rated their agreement about their understanding of the AI system in four 5-pointLikert scale statements (see Table 2).

### Results

**Fig. 2:**
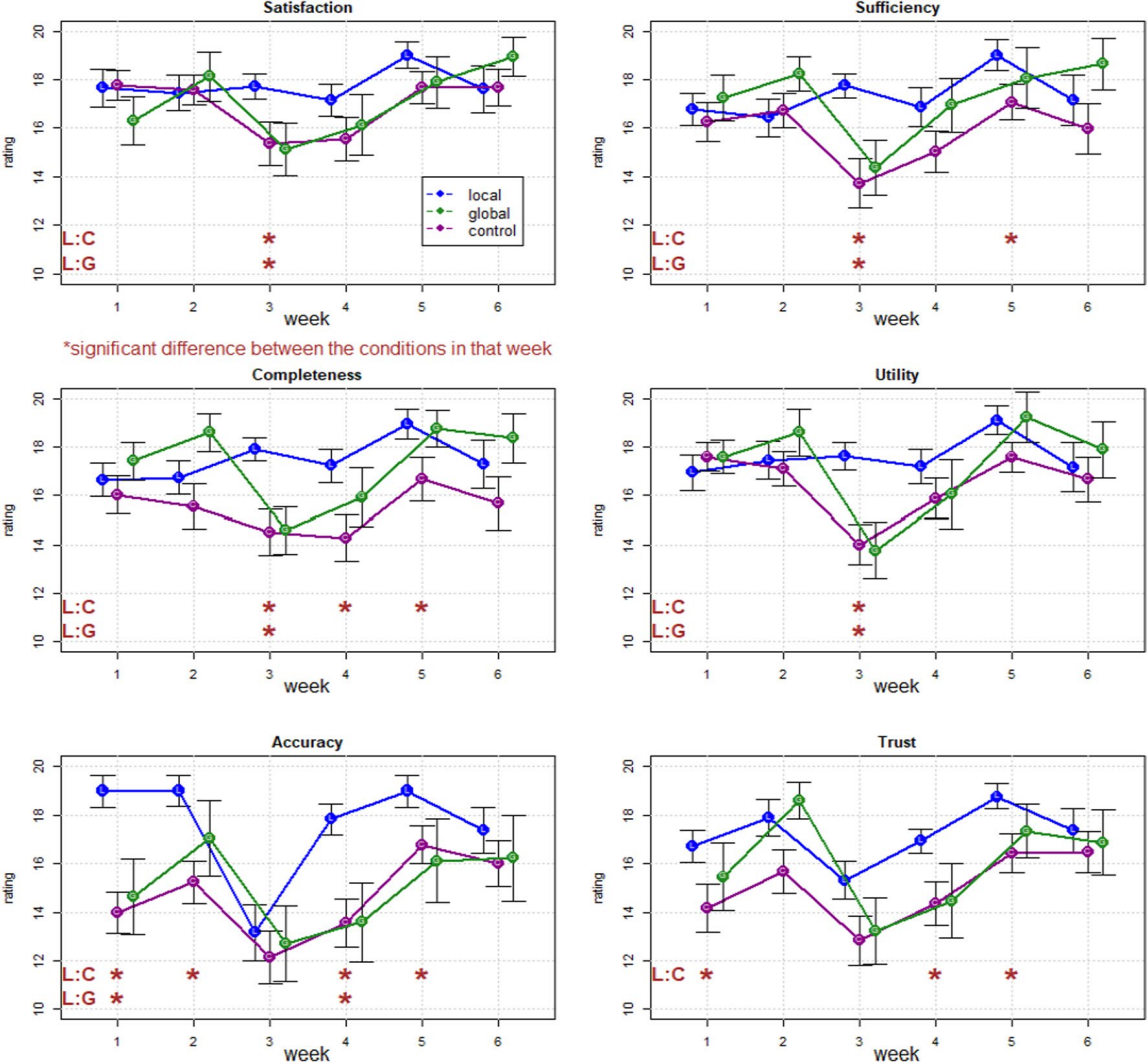
Results for explanation satisfaction scales

Both the control and the global explanation groups expressed less satisfaction, trust, perception of accuracy, sufficiency, usefulness, and completeness than the local explanation group, as shown in Fig. 2 (Rating scale was 1–21, but the figure shows 10–20 range for clearer visual). As a rough assessment of difference, we mark pairwise differences at each week that were significant at the *p* < 0.05 level according to a paired-samples t-test with a “*”.

The control group and global explanation groups received the same scenario with no local explanations, and only differed in whether they saw an initial global explanation of the AI, and so the fact that they did not differ from one another on these ratings suggest that the satisfaction ratings focus the user on the immediate situation and are not impacted by global understanding.

We examined the rating for each dimension of explanation satisfaction scales with a Type-III factorial ANOVA examining the main effects of time, explanation condition (local, global, and control), and their interaction using the R package ‘*ez’* [65]. The Type-III ANOVA examines the main effects AFTER the interaction has been accounted for, allowing us to identify residual effects of explanation types across all time points. The results are shown in Table 1.

**Table 1:** Results from Type-III factorial ANOVA for explanation satisfaction scales (n = 80)

	Time (week)	Explanation	Explanation: time
Satisfaction	*F* (5,385) = 8.20 *p* < 0.001 η_p_^2^ = 0.04	*F* (2,77) = 0.54 *p* = 0.58 η_p_^2^ = 0.01	*F* (10,385) = 2.28 *p* = 0.01 η_p_^2^ = 0.02
Sufficiency	*F* (5,385) = 7.52 *p* < 0.001 η_p_^2^ = 0.03	*F* (2,77) = 1.63 *p* = 0.20 η_p_^2^ = 0.03	*F* (10,385) = 3.14 *p* < 0.001 η_p_^2^ = 0.03
Completeness	*F* (5,385) = 6.20 *p* < 0.001 η_p_^2^ = 0.03	*F* (2,77) = 2.95 *p* = 0.06 η_p_^2^ = 0.04	*F* (10,385) = 2.28 *p* = 0.01 η_p_^2^ = 0.02
Usefulness	*F* (5,385) = 11.27 *p* < 0.001 η_p_^2^ = 0.06	*F* (2,77) = 0.95 *p* = 0.39 η_p_^2^ = 0.01	*F* (10,385) = 2.83 *p* = 0.002 η_p_^2^ = 0.03
Accuracy	*F* (5,385) = 17.40 *p* < 0.001 η_p_^2^ = 0.08	*F* (2,77) = 4.16 *p* = 0.02 η_p_^2^ = 0.06	*F* (10,385) = 2.18 *p* = 0.02 η_p_^2^ = 0.02
Trust	*F* (5,385) = 13.31 *p* < 0.001 η_p_^2^ = 0.07	*F* (2,77) = 3.03 *p* = 0.05 η_p_^2^ = 0.04	*F* (10,385) = 0.87 *p* = .0.50 η_p_^2^ = 0.01

Time and explanation refer to the main effects and Explanation: Time refers to the interaction between these independent variables

All interactions of Time and Explanation were significant at the *p* < 0.05 level except for the trust response. The tests also showed the main effects of explanation were statistically significant for accuracy indicating that it was deemed better for explanation conditions across the entire duration of the experiment. Finally, the main effects of time were seen for all measures, indicating that the scenario was potent enough to manipulate subjective measures of trust as it moved through initial diagnosis to rediagnosis to resolution.

Welch t-test was conducted for comparing each pair of explanations at each week, the local explanation group with the control group, local explanation group with a global explanation group, and global explanation with the control group. The significant differences between each pair at each week are shown in Fig. 2 with a “*”.

**Fig. 3:**
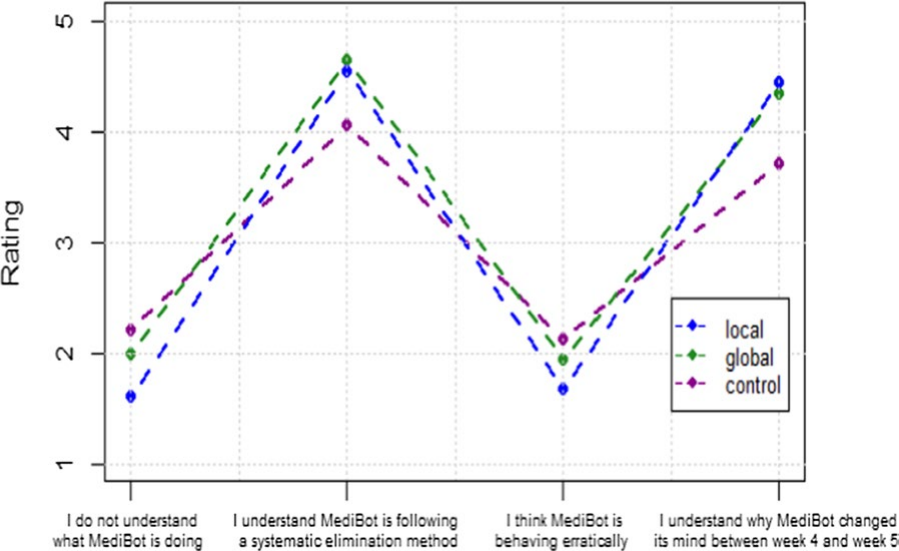
Results from statement ratings

In contrast to the satisfaction ratings, the ratings of understanding elicited at the end of the scenario did in fact lead to differences between global explanation and the other conditions (see Fig. 3). A one-way ANOVA showed that the three explanation conditions were significantly different (*p* < 0.05) for the statements “I understand MediBot is following a systematic elimination method” (*F* (2,77) = 8.7, *p* < 0.001) and “I understand why MediBot changed its mind between week 4 and week 5” (*F* (2,77) = 8.3, *p* < 0.001), but they were not significantly different for the statements “I do not understand what MediBot is doing” (*F* (2,77) = 2.6, *p* = 0.08) and “I think MediBot is behaving erratically” (*F* (2,77) = 2.3, *p* = 0.11).

We used a post-hoc Tukey test at a *p* < 0.05 significance level on the three groups to examine pairwise differences (see Table 2). We used the TukeyHSD function in Rstudio for the post-hoc tests that only return the significance value for post-hoc tests and not any specific statistics. The global explanation condition produced ratings that were significantly better than the local explanation for statements 1 and 2, and both the local and global conditions were rated better than the control for statements 2 and 4. There were no differences between groups on statements 1 and 3. Thus, although the initial global explanation was not helpful for improving satisfaction during the scenario, it provided a better overall understanding of the general method of diagnosis by the AI system.

**Table 2:** Post-hoc Analysis for final understanding

	Control- local Ex	Control-global Ex	Local–global
1. I do not understand what MediBot is doing	*diff* = 0.61 *p* = 0.06	*diff* = 0.22 *p* = 0.77	*diff* = 0.39 *p* = 0.45
2. I understand MediBot is following a systematic elimination method	*diff* = *−0.49* *p* = 0.002	*diff* = −0.6 *p* = 0.002	*diff* = *0.1* *p* = 0.83
3. I think MediBot is behaving erratically	*diff* = 0.45 *p* = 0.09	*diff* = 0.18 *p* = 0.74	*diff* = 0.26 *p* = 0.55
4. I understand why MediBot changed its mind between week 4 and week 5	*diff* = − 0.73 *p* < 0.001	*diff* = − 0.63 *p* = 0.02	*diff* = − 0.1 *p* = 0.9

Each pair-wise comparison (n = 80) was performed with a pairwise Tukey HSD test

### Discussion and summary

#### Impact of local explanation/justification

In this study, we examined how a re-diagnosis event impacted satisfaction and trust, and how different kinds of explanations impacted satisfaction, trust, and understanding of an AI system. Overall, the study showed that satisfaction and trust are harmed at the critical points during rediagnosis, even when the system is making the best diagnosis based on available information. Interestingly, the global explanation, which attempted to inoculate participants by teaching them that this very situation might occur, did little to reduce the impact of the rediagnosis on immediate measures of satisfaction and trust. Local justifications had effects throughout the scenario, but their greatest effect was at the point of rediagnosis, in which they typically prevented a significant decline in subjective ratings of trust and satisfaction; and maintained this higher level of satisfaction until the end of the scenario when the diagnosis was resolved.

Thus, we found that local justifications were effective, but their effect is time sensitive. During a critical situation or when AI was making errors, local justifications were very effective and powerful explanations for the patients.

#### Impact of global explanations

In contrast, pre-test global explanations using example diagnoses do not show the same benefits. The global explanation did not help to raise satisfaction measures during the diagnosis in comparison to the control group that received no explanations. However, the global explanation brought significant changes to the perception of the overall understanding of the AI system.

This study shows an initial demonstration of the time course of trust, satisfaction, and understanding during an unfolding diagnostic scenario. In the study, we used very simple visual explanations—bar charts describing the probabilities of different outcomes, with accompanying text. It is important to note that these explanations appeared effective, even though they are much simpler than many current explanatory algorithms that have been proposed for similar situations.

There are a number of alternative methods that have been explored for the explanation of classification and diagnosis. One approach attempts to focus attention on important causal factors in a classification decision or diagnosis [66]. Although like our study, the relative likelihood of different outcomes is typically shown, algorithms also often try to identify the importance of different features in making the diagnosis. For example, in the IBS/Celiac scenario, a symptom of joint pain supported celiac better than IBS, but not enough to override the higher base rate of IBS (especially because joint pain could arise from other sources and thus be attributed to something else). A single test for a gluten allergy would have been sufficient to change the diagnosis from the higher-base-rate IBS to the low-base-rate Celiac but could not impact the diagnosis if the test is not run. It might be important to let the patient understand how different signs and symptoms feed into the overall diagnosis.

A second method for explanation has been to rely on judiciously chosen examples. Examples and cases are known to be important methods for reasoning and persuasion [29, 30, 67] and have been extensively explored in the XAI literature [45].

To understand how more complex explanations might impact satisfaction and trust, we conducted a second study using a similar diagnosis scenario to investigate how different forms of local explanations affect patient satisfaction, trust, and perception of accuracy during diagnosis, which we will report next. The first study was mostly exploratory, and the second study was designed to test more specifically what we found in the first one. In this second study, we will examine and compare feature-highlighting approaches with case-based approaches.

## Experiment 2: Exploring local explanation

Experiment 1 established that combining a logical rationale and a visual depiction of the probability distribution across outcomes provided some benefits to subjective assessments of satisfaction. Along with these components of explanation, XAI systems also use examples (both positive and contrasting) to help a user understand why a decision was made. The goal of this study was to investigate whether these different forms of explanation in an AI diagnostic system affect patient satisfaction, trust, and perception of accuracy. We implemented three forms of explanation: written rationales, visuals + rationales, and examples + rationales, in a diagnosis scenario similar to the one in Experiment 1. Again, a simulated AI system gave a most likely but incorrect diagnosis, but later it changed the diagnosis to the correct disease. We hypothesized that each of the explanation types would provide benefits over control, and (as demonstrated in Experiment 1), they would do so primarily at the critical rediagnosis point. Furthermore, we hypothesize that adding additional information to written rationales (in the form of example or visualizations) may provide an additional benefit because they may provide the information in a more comprehensible way, and so we will test whether either produces a benefit over rationale alone.

### Method

#### Participants

One hundred and thirteen undergraduate students at Michigan Technological University took part in the study in exchange for partial course credit. No participant from the first experiment participated in this experiment but the population was similar to the first experiment.

#### Procedure

The study was conducted online, and it took 15–20 min to complete. Participants gave their consent online before taking part in the study. They played the role of a patient suffering from a gastrointestinal disorder interacting with the simulated AI system slightly modified from the Experiment 1 scenario.

This time, the simulated patient suffered from abdominal pain, cramps, bloating, diarrhea, fatigue, and joint pain and had no family history of gastrointestinal diseases but had recently been exposed to a natural water source, making an initial diagnosis of Giardia likely. When tests for this came back negative, MediBot predicted that it might be IBS and asked to follow the IBS diet. The patient’s condition was inconsistent for a few weeks following the diet, then eventually MediBot resolved the diagnosis as Celiac disease and confirmed it with tests.

Participants were randomly assigned into one of four groups, each receiving a different form of explanation: (1) text rationales as an explanation; (2) visuals + rationales explanation; (3) examples + rationales, and (4) a control group.

**Fig. 4:**
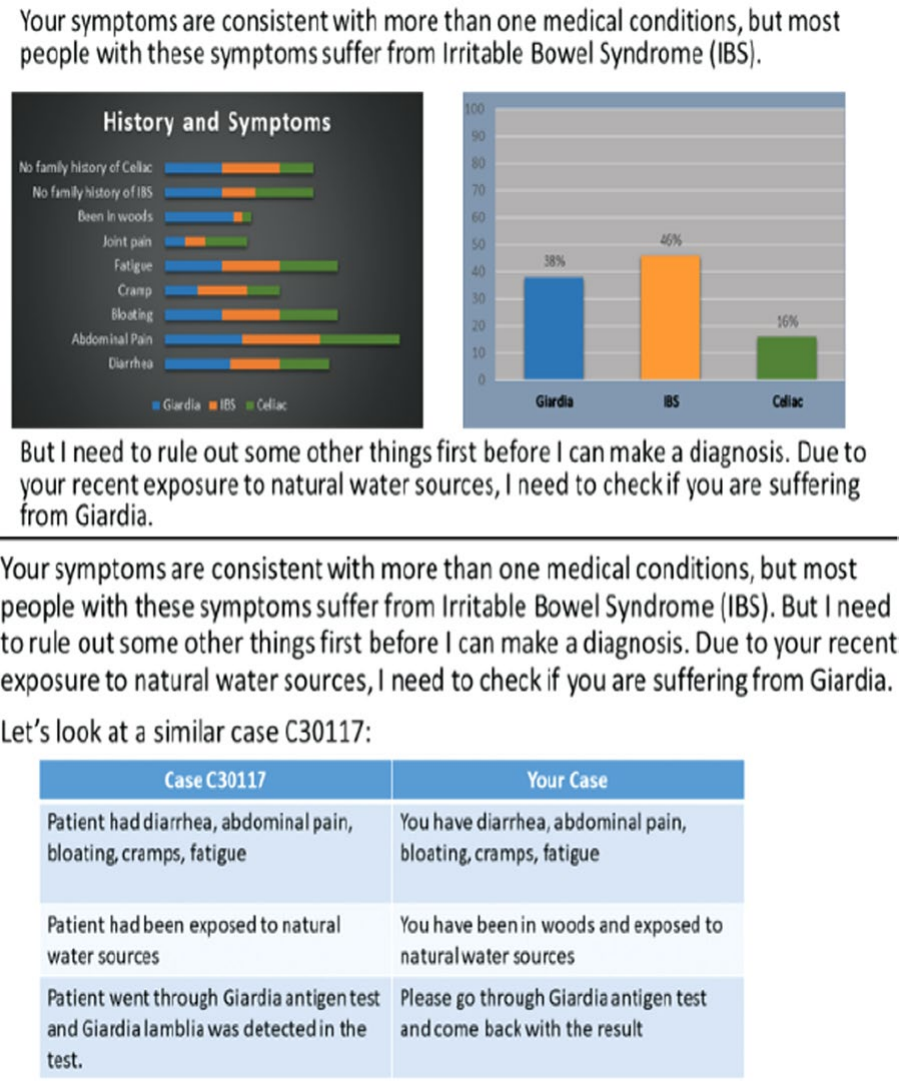
Sample explanations used in Experiment 2. Top panel shows visualizing feature weights and rationale; bottom panel shows example-based explanations

Rationales are the narrative justifications of how MediBot made decisions. Visual explanations include figures of the likelihood of each suspected disease based on features MediBot used to make decisions as shown in the top panel of Fig. 4. These visualizations were akin to the LIME algorithm [63] but were generated via a simple probabilistic Bayesian model (the symptom likelihood visualizations were given by the conditional probability of each disease given the symptom). We also showed the equivalent probability chart provided in Experiment 1, which showed the relative probability of each disease.

The example-based explanation included examples of similar cases diagnosed by MediBot in the past, as illustrated in the bottom panel of Fig. 4. We used example-based explanations where the system gave an example of a previous case and explained how it was diagnosed.

In week 5, instead of showing a positive example, it used an example that explains why it did not consider Celiac disease the most-likely condition at the beginning of the consultation. The rationales-only group saw all the justifications included in the visual and example-based explanation, only the figures and examples were removed from the explanation.

As in Experiment 1, participants interacted with MediBot for six simulated weeks and received an explanation about its prediction and diagnosis each week. After each simulated week, participants were asked to rate their satisfaction, trust, perception of accuracy, sufficiency, usefulness, and completeness for the explanations, as in Experiment 1 but this time they rated the measures using 7-point Likert scale.

### Results

**Fig. 5:**
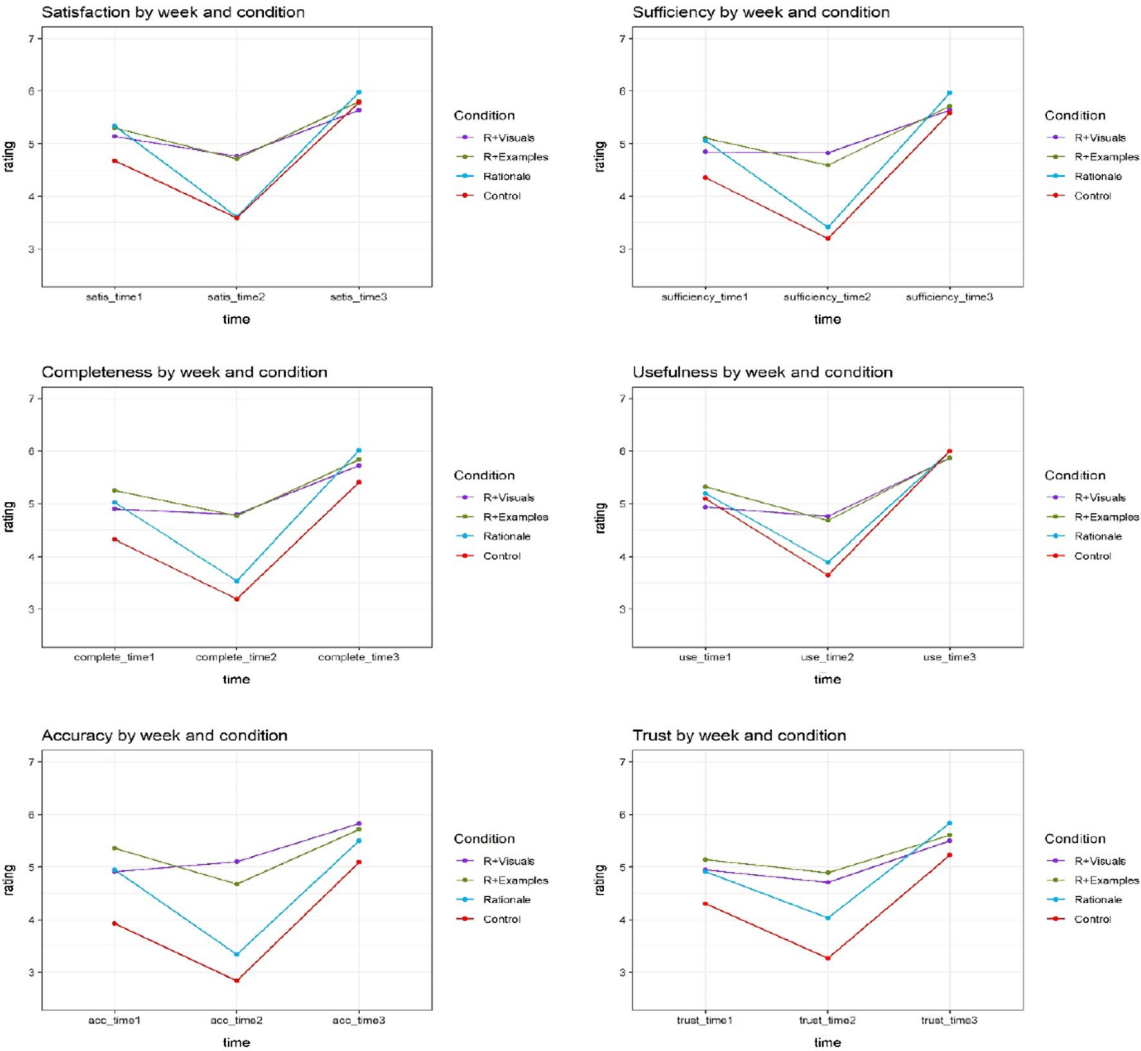
Rating for explanation satisfaction scales

Since we had a challenging multi-comparison analysis with six DVs (satisfaction, trust, perception of accuracy, sufficiency, usefulness, completeness) and 4 conditions (control, rationales, visuals + rationales, examples + rationales), we decided to organize the ratings for all six weeks into three sets: Week 1 and 2 averaged into Time 1 (initial diagnosis), Weeks 3 and 4 averaged into Time 2 (critical rediagnosis) and Weeks 5 and 6 averaged into Time 3 (resolution of diagnosis). The mean rating for all six attributes (satisfaction, trust, perception of accuracy, sufficiency, usefulness, and completeness) across conditions are shown in Fig. 5 (7-point Likert was used for ratings, but the figure shows 3–7 range for clearer visual).

We examined the rating for each dimension of explanation satisfaction scales with a Type-III factorial ANOVA examining the main effects of time, explanation condition, and their interaction using the R package ‘*ez*’ [65]. The Type-III ANOVA examines the main effects AFTER the interaction has been accounted for, allowing us to identify residual effects of explanation types across all time points. The results are shown in Table 3. The test of the interaction is the primary indicator of the effectiveness of an explanation because different conditions began with little difference and converged by the end of the study.

**Table 3:** Results from Type- III factorial ANOVA for explanation satisfaction scales (n = 113)

	Time (week)	Explanation	Explanation: time
Satisfaction	*F* (2,218) = 126.52 *p* < 0.001 η_p_^2^ = 0.25	*F* (3,109) = 1.97 *p* = 0.12 η_p_^2^ = 0.04	*F* (6,218) = 8.01 *p* < 0.001 η_p_^2^ = 0.06
Sufficiency	*F* (2,218) = 114.65 *p* < 0.001 η_p_^2^ = 0.25	*F* (3,109) = 3.38 *p* = 0.02 η_p_^2^ = 0.06	*F* (6,218) = 8.78 *p* < 0.001 η_p_^2^ = 0.07
Completeness	*F* (2,218) = 104.24 *p* < 0.001 η_p_^2^ = 0.24	*F* (3,109) = 4.85 *p* = 0.003 η_p_^2^ = 0.08	*F* (6,218) = 6.54 *p* < 0.001 η_p_^2^ = 0.06
Usefulness	*F* (2,218) = 110.36 *p* < 0.001 η_p_^2^ = 0.25	*F* (3,109) = 0.82 *p* = 0.49 η_p_^2^ = 0.02	*F* (6,218) = 5.06 *p* < 0.001 η_p_^2^ = 0.05
Accuracy	*F* (2,218) = 88.26 *p* < 0.001 η_p_^2^ = 0.20	*F* (3,109) = 9.95 *p* < 0.001 η_p_^2^ = 0.16	*F* (6,218) = 8.14 *p* < 0.001 η_p_^2^ = 0.07
Trust	*F* (2,218) = 64.71 *p* < 0.001 η_p_^2^ = 0.16	*F* (3,109) = 4.71 *p* < 0.001 η_p_^2^ = 0.08	*F* (6,218) = 4.10 *p* < 0.001 η_p_^2^ = 0.04

Time and explanation refer to the main effects and Explanation: Time refers to the interaction between these independent variables

All interactions of Time and Explanation were significant at the *p* < 0.05 level. The tests also showed the main effects of explanation were statistically significant for sufficiency, completeness, accuracy, and trust indicating that these were deemed better for explanation conditions across the entire duration of the experiment. Finally, the main effects of time were seen for all measures, indicating that the scenario was potent enough to manipulate subjective measures of trust as it moved through initial diagnosis to rediagnosis to resolution.

To understand the differences between the Explanation Conditions at each time set, we conducted Tukey post-hoc tests for each of the six scales using the R package *agricolae* [68]. The results are shown in Table 4.

**Table 4:** Significant differences between conditions (n = 113) at each Set according to the Tukey test, any pairing not mentioned was not significantly different for that Set

	Time 1	Time 2	Time 3
Satisfaction	None	Visual; examples > rationale; control	None
Sufficiency	None	Visual; examples > rationale; control	None
Completeness	None	Visual; examples > rationale; control	None
Usefulness	None	Visuals were better than control	None
Accuracy	Example > control	Visual; examples > rationale; control	None
Trust	None	Visuals; examples > control	None

For Time 1, there are no significant differences between any pair of explanation types overall six dimensions except accuracy. At Time 2, there are no significant differences between visuals + rationales and examples + rationales for satisfaction, sufficiency, completeness, trust, and accuracy. But they both were better than control and rationales for satisfaction, sufficiency, completeness, and accuracy. At Time 3, there are no differences between any of the explanation types, indicating that the resolution of the scenario produced uniformly high satisfaction. Only during the rediagnosis crisis weeks, when the system was noticeably wrong, there were statistically significant differences between explanation conditions.

**Fig. 6:**
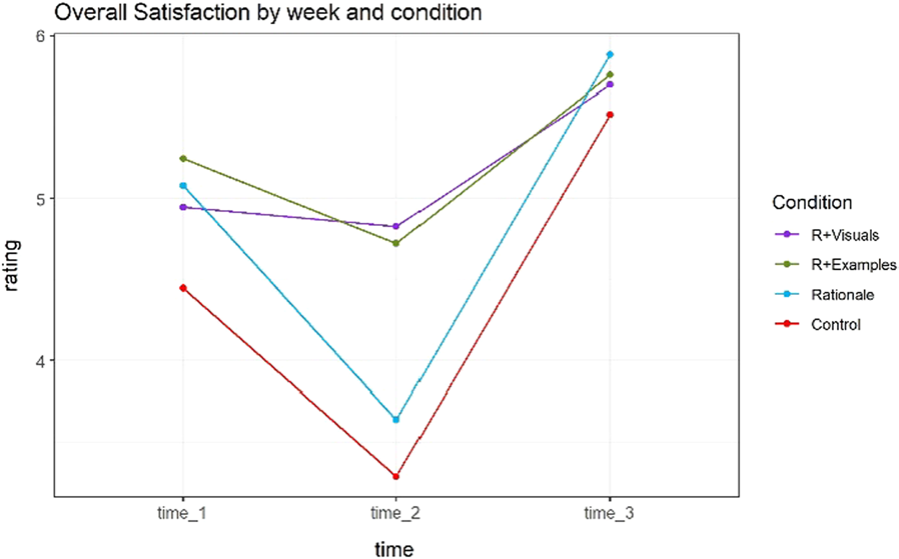
Mean rating for Overall Satisfaction

Figure 6 summarizes these 6 measures with a single grand average that encapsulates the basic effect of explanation conditions in our scenario. A Type-III factorial ANOVA on the average score showed a statistically significant difference in overall satisfaction by time (*F* (2,218) = 144.68, *p* < 0.0001), explanation (*F* (3,109) = 4.43, *p* = 0.006) and the time by explanation interaction (*F* (6,218) = 9.14, *p* < 0.001).

A post-hoc Tukey test showed there were no significant differences between any pair of explanation types at Time 1; there are no significant differences between visuals + rationales and examples + rationales but they both are better than control and rationales at Time 2, and there were no differences between any of the explanation types at Time 3.

To investigate this in detail, we did a separate analysis for comparing (1) rationale vs. control; (2) rationale vs. visuals + rationales; and (3) rationale vs. examples + rationales. This maps onto our hypothesis as well. A Type-III factorial ANOVA examined the main effects of time, explanation condition, and their interaction. The main effects of explanation were not statistically significant for neither of them: rationale vs. control (*F* (1,54) = 3.4, *p* = 0.07), rationale vs. visuals + rationales (*F* (1,55) = 1.22, *p* = 0.27), and rationale vs. examples + rationales (*F* (1,54) = 2.37, *p* = 0.13). The main effects of Time were significant at the *p* < 0.05 level for rationale vs. control (*F* (2,108) = 132.42, *p* < 0.001), rationale vs. visuals + rationales (*F* (2,110) = 85.12, *p* < 0.001), and rationale vs. examples + rationales (*F* (2,108) = 66.97, *p* < 0.001). Interactions of Time and Explanation were statistically significant at the *p* < 0.05 level both rationale vs. visuals + rationales (*F* (2,110) = 21.16, *p* < 0.001) and rationale vs. examples + rationales (*F* (2,108) = 9.72, *p* < 0.001) but not for rationale vs. control group (*F* (2,108) = 0.66, *p* = 0.52).

### Discussion and summary

This study demonstrated several important results. First, like Experiment 1, explanations only appear to matter substantially during crisis weeks. It must be noted that this crisis was not due to a specific mistake or error on the part of the AI, but it was a consequence of making a most-likely diagnosis based primarily on the relative base rate of two diseases that have similar symptomology. Second, we found that richer explanations (visuals + rationales and examples + rationales) are the most effective at these critical points, but otherwise do not differ substantially from the control group. Next, for the majority of measures, rationales alone were no better than the control group. Additionally, although the visualization was substantially different from example-based explanations, we found no evidence that one method was more effective than the other. Finally, once the system came to a resolution the explanation no longer mattered and participants gave high satisfaction ratings.

Notably, this experiment did not test several conditions that might also be interesting. First, because of the lack of impact of global explanation on satisfaction measures, we did not compare global explanations in this study, either alone or accompanying the local explanations. We have no data on whether the global explanation would improve local justifications in this scenario but suspect that they would have little impact here as well. We also did not examine whether together, examples and visualizations would be better than either individually. The fact that subjective ratings improved at Time 3 versus Time 2 shows that there would certainly be room for improvement in the score but given that neither were substantially impaired during Time 2 in comparison to the baseline Time 1 suggests that satisfaction may be as high as it can be under the circumstances of a disease that has not yet been cured. Finally, we examined only a single method of selecting examples. This method was sufficient to increase self-rated satisfaction of the system, but it may be the case that there are a variety of example types that could provide better or worse explanations. Our examples were chosen specifically to provide similar cases in the past that produced similar outcomes; another approach would be to use contrastive examples that highlight a critical aspect of the symptoms that led to the current diagnosis.

## General discussion

The two studies reported here allow us to draw several conclusions about how patient-facing explanatory diagnostic systems may succeed or fail. Overall, they show the importance of context on explanations. For example, justifying a decision is important to maintain satisfaction in the system; different kinds of explanations impact the patient differently, and the timing of explanations is also critical. We will examine the main lessons from these studies next and provide some recommendations for existing AI diagnostic systems.

### Lesson: explanations are time-sensitive

These studies found that explanations are differentially effective at different timepoints. At the critical times when the AI is making errors, explanations can be very helpful for improved patient satisfaction, whereas they were often no different from control when things at non-critical points. This suggests that to manage patient attention and focus, developers may wish to avoid burdening patients with explanations when none are needed. Not only can this be distracting, but an explanation for something that is already understood may make the patient think they misunderstand something (why else would it need to be explained). Consequently, explanations should be used judiciously at appropriate times.

The impact of explanations at the critical Time 2 is important because this is the point at which real patients might start abandoning the system, seeking second opinions, or failing to adhere to recommendations. The type of error seen in this scenario is especially pernicious because the diagnosis was in some sense optimal, even though it is wrong. The study shows that under the right circumstances, an explanation may mean the difference between seeing this and thinking that the diagnosis system is fundamentally unreliable or inaccurate.

### Lesson: significance of global explanation

Global explanations were not as effective as local justifications for immediate measures of patient satisfaction and trust. Nevertheless, they showed significant improvement in some post-scenario measures—ones related to the perception of global or overall understanding of the diagnosis by the AI system. And so, that should not be ignored if developers are really trying to build an XAI system for the patients. Thus, not only are different explanations effective at different times, but they also impact different aspects of their assessment of the system.

### Lesson: effectiveness of local justification

These studies showed the power of local justification/explanation on immediate measures of satisfaction. When used at the right time, a local justification could be a powerful improvement for diagnostic systems. Our results suggest that system developers should concentrate on investing more effort into explanations in cases where the system may be wrong, and especially when a diagnosis is changing. Most XAI systems currently focus on a single time point explanation, but if a system can detect that its predictions are changing in a single case, this is an especially important point to use explanation.

### Lesson: the format of explanation matters

Across the two studies, we examined several different formats of explanation. We found that even a simple visualization showing the likelihood of different outcomes was effective (Exp. 1), as were more complex visualizations (Exp. 2) and examples (Exp. 2). However, a written logic-based narrative explanation alone (Exp. 2) did not improve subjective assessments of satisfaction. Not only that, but a detailed global explanation anticipating the type of mistakes the AI would make had its greatest impact on post-scenario ratings of knowledge and not immediate measures of satisfaction. There are various forms of visual explanations and case-based or example-based explanations offered in XAI literature. Our studies suggest that instead of asking simple comparisons about “which kind of explanation is better”, researchers should start addressing questions about when and how different kinds of explanations are effective and helpful.

### Lesson: diagnosis is not simply classification

One final observation we make is that it is a mistake to think about AI diagnosis as merely a classification problem that determines a disease or condition based on symptoms and signs. For example, Alam [44] identified how diagnosis involves explaining why and how the AI is making the diagnosis. As in our scenario, an error is not necessarily an actual mistake, it might be the most likely outcome that happens to be wrong for an individual. In other cases, the course of treatment may not simply be following the most-likely option. Instead, a treatment (e.g., antibiotics) may be pursued even if it is not the most likely if it has little risk, but the consequences of not treating it are large. Moreover, the present studies show that the necessary explanations depend on an evolving time course of diagnosis, and explanation is likely to interact with this timeline, as it may help surface information the physician did not previously know.

### Recommendation for existing AI diagnostic systems

Based on the main lessons learned from our studies, we have several recommendations for existing AI diagnostic systems using the healthcare chatbot “Ada” as a representative example to show how it could be improved based on our findings. Ada offers an AI-powered health and symptom assessment application that helps its users to understand their health condition and navigate to the appropriate care [69]. One possible improvement is to maintain persistent awareness of symptom tracking and change. Though Ada tracks symptoms after it provides possible causes of the symptoms of a user, it does not provide any recommendation or explanation if the symptoms worsen or persist and does not track if there are any new symptoms either. Symptom change represents critical points at which explanations are important. At such critical points, it should be able to explain to the user why this is happening.

A second improvement would be to incorporate global explanation about the diagnostic strategy. Although Ada provides some local visual justifications while presenting the likelihood of the possible causes, it does not provide any explanations about how it makes decisions in general. Such global explanation may help users understand the overall decision-making process. Third, more information about alternative outcomes and diagnoses seems to be important. Ada provides a full assessment report of the symptoms showing how many people out of 10 people with similar symptoms might have some medical condition, but it only focuses on a single time point. It could incorporate what may happen if any of the symptoms change over time and provide some example cases as explanations for the users. Finally, explanations via example cases can be useful, especially to highlight variety and contrasting outcomes.

## Limitations

This study was designed to test several general explanation mechanisms in a hypothetical diagnostic scenario, tested on a relatively inexperienced and homogenous college population. This somewhat limits our ability to generalize to conclusions about how an older, less educated population might have responded in these scenarios or an older population with more experience dealing with health care or gastrointestinal disorders. This is a limitation of our approach, but it is important to acknowledge that the diagnostic systems we are simulated do not exist, and our participants were not really suffering from the disorder, but we believe that at the current state of prospective development, even rough assessments of satisfaction can be valuable. Certainly, our results may exhibit some differences if there were multiple complex scenarios or cases with a specific target population e.g., older adults, patients of rare or chronic diseases. Such a subject population may interpret these explanations differently from what we found in our study. Our studies may provide a guideline for future research that will involve user evaluation of real-world patient-facing AI diagnostic systems, where it will be useful to know what kind of explanations are effective for their trust, satisfaction, and understanding of these systems. Furthermore, the experiments serve as a baseline for validating the scenario and the satisfaction measurements to establish that together, they can create a situation in which satisfaction is sensitive to explanation. Future research is needed to establish whether these scenarios and measures will generalize to other populations.

Another limitation of this work involves the extent to which measures of satisfaction and trust of a simulated patient in our scenario matter, given the fact that they are likely to have very limited knowledge of the diagnostic problem before using a hypothetical diagnostic system. Currently, a patient might consult WebMD or other on-line sources to find possible diagnoses that map onto symptoms they are experiencing. This may help them understand the different possibilities and may even allow them to try different non-medical treatments (changes of diet, etc.) without consulting a physician. Yet they are unlikely to be able to legitimately assess the trustworthiness of the system because they do not have the knowledge of a physician with an understanding of both biological mechanisms and the likelihood of different diagnoses. Whether patient satisfaction is related to the accuracy of the system, it is likely to influence adoption and abandonment, and so care must be taken to ensure that these measures of satisfaction and trust are not interpreted as related to the accuracy of the system (which [64] distinguished as relating to performance).

## Conclusion

To improve patient satisfaction and trust at such points, building AI systems with higher accuracy might not be enough, and may not even be possible. In critical situations, AI systems may offer an erroneous diagnosis in the process of determining the most-likely disease or condition, but patients would not understand the reason behind this if they do not get exposed to the explanations and justifications. Incorporating appropriate explanations with the AI systems may help a patient understand the diagnosis better in these situations and make them satisfied with the diagnosis as well.

## Supplementary Information

**Additional file 1**. Supplementary-Appendix.

## Data Availability

The datasets used and/or analyzed during the current study are available from the corresponding author on reasonable request.
