# Supplementary material for: Examining the effect of explanation on satisfaction and trust in AI diagnostic systems
Source: BMC Med Inform Decis Mak. 2021 Jun 3;21:178. doi: 10.1186/s12911-021-01542-6 (PMC8176739; doi:10.1186/s12911-021-01542-6)
Supplement: Supplementary file 1 — Additional file 1. Supplementary-Appendix. [file 12911_2021_1542_MOESM1_ESM.docx]

The Myth of Diagnosis as Classification: Examining the Effect of Explanation on Patient Satisfaction and Trust in AI Diagnostic Systems

Lamia Alam^1^, Shane Mueller^2^

Michigan Technological University, Houghton, MI 49931, USA
[lalam@mtu.edu^1^](mailto:lalam@mtu.edu1) shanem@mtu.edu^2^

Appendix

The scenario for Experiment 1:

| Week No. | Decision of MediBot |
| --- | --- |
| 1 | The patient consults with MediBot for diarrhea, abdominal pain, cramps, fatigue, and joint pain. MediBot suspects it is IBS, it advises to follow a strict diet chart and come back next week. |
| 2 | Patient feels better after following the diet chart, MediBot advises to continue the diet chart and come back next week. |
| 3 | Patient again starts feeling worse suffering from diarrhea, bloating, fatigue, and joint pain. MediBot thinks that the IBS diet chart takes some time to adjust with the body and joint pain might occur due to arthritis. It advises to continue the diet chart and come back next week. |
| 4 | Patient’s condition is not improving. MediBot changes its mind and the recurrence of the symptoms lead it to consider that patient might be suffering from some other condition. It asks patient to go through some tests and come back with the report next week. |
| 5 | Patient comes back with the report and it is found that patient is suffering from Celiac disease, which occurs due to gluten allergy. Celiac has similar symptoms like IBS but shows some additional symptoms like joint pain and fatigue. MediBot asks the patient to consult a nutritionist to follow a gluten-free diet and come back for a follow up next week. |
| 6 | Patient feels great due to following a gluten-free diet. |

The scenario for Experiment 2:

| Week No. | Decision of MediBot |
| --- | --- |
| 1 | The patient consults with MediBot for diarrhea, abdominal pain, bloating, cramps, fatigue, and joint pain. Patient has been exposed to natural water source because of hiking/camping. MediBot suspects it is Giardia, it asks patient to go through Giardia antigen test. |
| 2 | Giardia antigen test comes negative, but the symptoms persist. So, Medibot suspects it is IBS, advises to follow a strict diet chart and come back two weeks later. |
| 3 | Patient feels better after following the diet chart, some symptoms are gone. MediBot advises to continue the diet chart and come back next week. |
| 4 | Patient again starts feeling worse suffering from diarrhea, abdominal pain, bloating, fatigue, and joint pain. MediBot asks the patient to cut gluten from the diet and come back next week. |
| 5 | Patient feels much better. MediBot changes its mind; the recurrence of the symptoms and the improved outcome of gluten-free lead it to consider that patient might be suffering from Celiac disease. It asks patient to go through some tests and come back with the report next week. |
| 6 | Test results indicate that the patient is suffering from Celiac disease. Patient is feeling great due to following a gluten-free diet. Medibot advises to visit a nutritionist to follow a proper diet. |
